# The Health Status of Undocumented Immigrants from Asian Countries in the United States: A Scoping Review and Recommendations for Future Directions

**DOI:** 10.1007/s10903-024-01625-2

**Published:** 2024-08-24

**Authors:** Sameera S. Nayak, Amanda Cardone, Kina Soberano, Meghan Dhond

**Affiliations:** Department of Sociology, Anthropology, and Public Health, University of Maryland Baltimore County, 1000 Hilltop Circle, Public Policy Building, Baltimore, MD 21250 USA

**Keywords:** Undocumented, Immigrant Health, Asian immigrants, Scoping review, DACA

## Abstract

Immigrants from Asian countries are the fastest-growing undocumented population in the United States (U.S.), yet not much is known about their health. This scoping review identifies the nature and extent of scientific literature on the health of undocumented Asian immigrants in the U.S. We conducted a comprehensive search of six electronic databases in 2024. Inclusion criteria were empirical articles written in English, published in peer-reviewed scientific journals from 2010 to 2024, and focused on a health outcome or health-related issue involving undocumented Asian immigrants. Results are summarized narratively. We identified 13 peer-reviewed publications. Nine studies were quantitative, and four were qualitative. Eight studies were conducted in California; two studies used national secondary data sources. Studies were mixed in their research focus. They covered a range of health outcomes and issues, such as mental health (n = 4), health services and access (n = 2), contraceptive use (n = 1), COVID-19 (n = 2), and HIV (n = 1). Three studies measured self-rated health alongside other conditions, such as disability, health insurance coverage, chronic health conditions, and obesity. Scholarship on the health of undocumented Asian immigrants is a growing research area. Given the small number of studies identified, future research with larger diverse samples, more robust methodology, and greater topical variety are warranted to understand the health of this population better and reduce potential inequities.

## Introduction

Immigration status, sometimes called legal status or documentation status, has been identified as an essential determinant of health for undocumented immigrants by influencing access to health services and resources and increasing vulnerability to immigration enforcement, exclusionary policies, and associated stress [1–4]. Legal status plays a significant role in shaping the health behaviors of undocumented populations [1, 4]. Undocumented Asian immigrants (UAIs) make up approximately 11% of the 11.2 million undocumented individuals in the United States (U.S.) [5]. Recent estimates place the number of UAIs at 1.2 million [5]; in 2021 the total population of all Asian immigrants was 14.3 million [6]. The share of the undocumented population from Asia who arrived within the last five years grew from 13 to 22% between 2007 and 2017 [7]. Likewise, between 2008 and 2021, the overall UAI population grew by 47% [5]. While studies on the health and wellness of Asian immigrants are abundant [22–27], only a small number have narrowed their focus to UAIs [28–30]. Many existing studies center on cultural differences and acculturation as drivers of health inequities for Asian immigrants [31–33]. Studies with other undocumented communities, such as Latinx communities, have found that undocumented status is associated with reduced healthcare access, increased prevalence of common mental health disorders such as depression and anxiety, trauma, increased risk of HIV, social exclusion, and reduced help-seeking after experiencing crime due to fears of detention and deportation, among others [8–18]. Research with undocumented African immigrants has identified complex barriers to health service access and discrimination within healthcare settings that contribute to disparities [19–21]. Whether UAIs face analogous health challenges and disparities as other undocumented groups due to their precarious legal status is not fully understood.

### Contemporary Experiences of UAIs

Most UAIs become undocumented by continuing to reside in the U.S. after their visa (e.g., student visa or high-skilled work visa) has expired [34, 35]. Therefore, unlike other groups who may have always been undocumented since their arrival in the U.S., UAIs often have some form of legal status prior to becoming undocumented. This might provide UAIs with protections if they become undocumented through this mechanism as these modes of entry are generally available to those with higher socioeconomic status and education levels. On the other hand, these mechanisms are generally utilized by adults, which means that UAIs who become undocumented through these routes may not have amassed strong local support systems or social networks. These UAIs might experience adverse health effects as a result of becoming undocumented due to fragmented transnational social networks, loss of access to health insurance and preventative health care, and loss of employment and educational opportunities. Immigrants from India and China are the largest UAI groups [36] and thus might be most impacted by these issues. These hypotheses are yet to be empirically tested. Another important UAI subgroup are those eligible for protection through the Deferred Action for Childhood Arrivals (DACA) program. DACA provides temporary legal status to some young adults who were brought to the United States as children [37].

### Brief History of Asian Immigration

Immigrants from Asia are a heterogeneous group with different immigration histories. Similar to other communities of color, structural racism and white supremacy have shaped Asian immigration to the U.S. [38–41]. Historically, discriminatory and exclusionary governmental actions such as the Chinese Exclusion Act of 1882, Immigration Act of 1917, the *Supreme Court U.S. v. Bhaghat Singh Thind* case of 1923, the Philippine Independence Act of 1934, and the internment of Japanese Americans in 1942, have denied people of Asian descent the privileges of U.S. citizenship, thus barring them from the rights and protections afforded to citizens [38]. The Immigration and Nationality Act of 1965 reversed some of these policies, putting an end to separate quotas based on race and ethnicity, and leading to a rise in immigration from Asian countries [42].

### The Intersection of Legal Status and Ethnic Differences

Diverse Asian immigrant subgroups also experience the convergence of racism and xenophobia differently. Over 50% of the global Muslim population resides in the Asia–Pacific region [43]. Muslim Asians in the U.S., whose migration histories predominantly stem from countries such as India, Pakistan, Bangladesh, and Indonesia, might face Islamophobia, racial profiling, and xenophobia [44]. For example, Muslim Asians and those perceived to be “Muslim-like,” such as South Asian Sikhs, were victims of hate crimes after the September 11th, 2001 terrorist attacks [45, 46]. East Asians and Southeast Asians have historically faced different forms of exclusion and discrimination through racist tropes such as “Yellow Peril” paired with legal discrimination through mechanisms such as the Chinese Exclusion Act of 1882. Likewise, many immigrants from Southeast Asian countries such as Vietnam and Cambodia were initially forced migrants through refugee and asylum programs, although this trend has changed in recent times [47]. Experiences of discrimination for these groups are further exacerbated by their status as refugees or asylees. More recently, East and Southeast Asians have experienced a rise in hate crimes and discrimination as a consequence of COVID-19-related xenophobia and Sinophobia [48, 49]. These issues point to the need to study Asian immigrant health through the intersecting axes of both racialization and legal status stratification. This approach aligns with other calls to better contextualize legal status as a racialized construct [50]. Studies on Asian immigrant ethnicities that fail to account for variation in legal status might miss important sources of additional stress. As an example, undocumented Muslim Asians might experience greater negative health effects from the compounding effects of both Islamophobia [44] and the anxiety and fear of being undocumented. These impacts might be attenuated in Muslim Asians who are naturalized or who have another form of secure legal status. More recently, country quota caps on lawful permanent residency (“green cards”) have resulted in extended wait times for Asian immigrants from India, China, and the Philippines [51]. Authorized immigrants from these countries face more barriers to permanent immigration to the U.S. and often have to wait for decades to obtain residency [52]. Studies that compare Asian immigrants by legal status, without paying attention to ethnic differences, might miss differing effects of racism. Existing research on UAIs has been hindered by small sample sizes in national datasets and a lack of disaggregated data, which make it challenging to study variation between diverse Asian ethnicities and those with different types of legal status [41]. There is thus a need for more research on the health and experiences of Asian immigrants who are undocumented.

### Current Study

This scoping review aims to identify and delineate the existing peer-reviewed literature on the health of UAIs. Scoping reviews are recommended for nascent research areas to map what is already known and provide directions for future research [53]. To our knowledge, this is one of the first comprehensive reviews describing the range and extent of research on the health of UAIs in the U.S.

## Methods

A literature search of published peer-reviewed literature was conducted using the *“Preferred Reporting for Systematic Reviews and Meta-Analyses—Extension for Scoping Reviews (PRISMA-ScR)”* guidelines [54]. Inclusion criteria were empirical research on the health (or on a health-related issue) of undocumented immigrants of Asian origin living in the U.S. Included articles had to be published in English and in a peer-reviewed academic journal as we were primarily interested in the scientific literature on this subject. Quantitative studies could be descriptive or analytic; they could include prevalence, incidence, and/or risk and protective factors related to a health outcome. Qualitative studies could include health and well-being perspectives from UAIs or individuals who serve the population. Studies on health-related issues such as insurance coverage, access to health services, barriers to health care, and utilization of health services were included. Studies that examined health interventions or that did not expressly focus on health (e.g., those focused on educational outcomes) were excluded. Studies of health interventions were excluded as assessing interventions is more appropriate for a systematic review. Studies conducted with Asian immigrants where we could not ascertain if participants were undocumented were excluded. Studies conducted with undocumented immigrants where we could not ascertain if participants were of Asian origin were also excluded. Non-peer-reviewed publications, books, dissertations, commentaries, reviews, editorials, and conceptual papers were excluded.

We searched PubMed, CINAHL, Web of Science, PsycINFO, Health Source: Academic/Nursing, and Social Work Abstracts in March 2024 for original research from 2010 to 2024. The search strategy included terms such as *Asian* and *undocumented* and *USA*, terms for Asian ethnic subgroups based on the U.S Census Bureau’s definition [55], as well as synonyms and other related terms (Example search: (Asian* or Japanese or Korean* or Chinese or Bangladeshi or Bhutanese or Cambodian* or Filipino* or Hmong* or Nepali or Nepalese or Pakistani* or Singaporean* or Sri Lankan* or Sinhalese or Taiwanese or Thai or Thais or Vietnamese or “from Asia” or “from Bangladesh” or “from Bhutan” or “from Cambodia” or “from China” or “from India” or “from Japan” or “from Korea” or “from Nepal” or “from Pakistan” or “from the Philippines” or “from Singapore” or “from Sri Lanka” or “from Taiwan” or “from Vietnam”) AND (“illegal” OR “undocumented” OR “unauthorized” OR “noncitizen” OR “alien” OR “illegal immigrant” OR “illegal alien” OR “undocumented immigrant”) AND (American* or “U.S.” or “in the US” or “in the USA” or “in America” or “in the U.S.A.” or “in the United States” or “US city” or “US cities” or “US state” or “US states” or “of USA” or “of U.S.A.”). Although this search strategy meant that studies unrelated to health were included in the initial search, this approach increased the likelihood that all possible studies on this topic were accurately identified. Search strategies were optimized for each database. Lastly, we used Google Scholar and hand-searched reference lists to ensure that all articles had been included. We did not identify any new articles using Google Scholar.

Two reviewers independently screened all records based on the title and abstract. Discrepancies between reviewers were resolved through discussion. The full-text review was split between two reviewers. A third reviewer [principal investigator] independently reviewed all full-text articles for inclusion. Consensus on the final articles to include in the review was achieved through discussion and arbitration by the principal investigator. Data were extracted into tabular format to collect study-specific information from included articles. One reviewer extracted the data from the texts for the tables and another reviewer reviewed the information to ensure accuracy. At least two reviewers participated in every stage of the search process.

We extracted data on the study type, source, aims, time frame, location, sample characteristics, the total number of participants, distribution of ethnic subgroups (if reported), and the primary health issues studied. Whenever possible, we used direct quotes in the tables to reduce the likelihood of misrepresentation or misinterpretation of each article’s findings. Given the small number of studies, results were not analyzed to make inferences regarding the health status of UAIs. Instead, we have presented the study aims and characteristics to provide an overall snapshot of existing research. The University of Maryland, Baltimore County Institutional Review Board classified this study as non-human subjects research and exempted it from review.

## Results

**Fig. 1:**
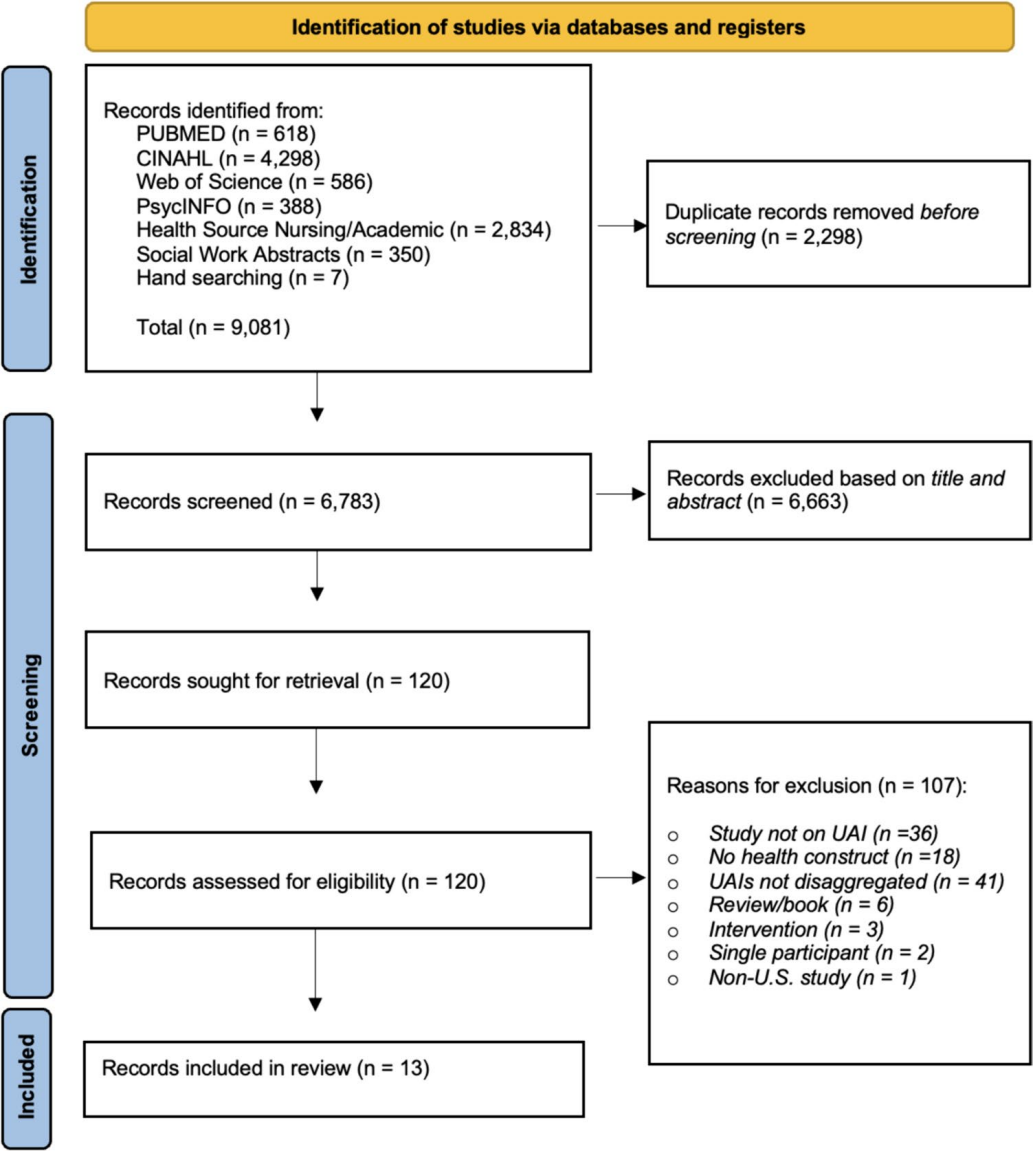
*PRISMA-ScR* flowchart of literature search and data collection process

We found 9,074 articles through database searching using the *PRISMA-ScR* guidelines. We identified seven additional articles through hand-searching reference lists. A total of 2298 duplicate records were removed. Figure 1 describes the search process [56]. Title and abstract screening were conducted for 6783 records, and 6663 records were excluded based on this. Reviewers assessed the full texts of 120 records. We excluded 107 articles for the following reasons: the study was not on UAIs (n = 36), UAIs were not disaggregated from other populations (n = 41), the study was not on health (n = 18), the study was on treatments or interventions (n = 3), the study was a review/book (n = 6), the study had a single participant (n = 2), the study was not in the U.S. (n = 1). A total of 13 articles were selected for final inclusion in this review. Of these, eight derived from Building community Raising API Voices for health Equity (BRAVE) studies [57]. Given that there might be overlap between samples from the articles using data from the BRAVE studies, we have presented each article separately for clarity and ease of interpretation.

### Primary Health Issues

Table 1 presents the research aims of the 13 studies in this review. We categorized studies based on the primary health issues they focused on. We have organized these based on the most common to the least commonly studied outcomes. Mental health outcomes were slightly more commonly studied (n = 4). Of these four studies, two focused only on depressive symptoms [30, 58], and two focused on mental health or psychosocial needs as well as health services and access [28, 29]. Self-rated health was measured in three studies [59–61]. Each of these three studies also measured other health-related issues such as health services and access [59], disability and health insurance coverage [61], chronic health conditions (asthma, diabetes, heart conditions, chronic bronchitis, hypertension, any type of cancer) and obesity [60]. There were two studies which focused on health services and access, including barriers and discrimination [62, 63]. Another two studies focused on COVID-19 [64, 65]. The remaining studies were on contraceptive use (n = 1) [66] and HIV (n = 1) [67].

**Table 1:** Summary of the Study Aims of the Articles in the Scoping Review

Authors	Aims^a^
Manalo-Pedro et al., 2022	“Estimate the effect of DACA status on clinical levels of depressive symptoms [and test] whether immigration enforcement experiences mediated this relationship [in undocumented Asian and Pacific Islander students]”
Ro, Nakphong, Choi, Nguyen, & Sudhinaraset., 2021	“Examine the role of social ties and depression among Asian and Pacific Islander undocumented young adults [and] distinguish between two types of social ties, bonding and bridging [and] the absence of social ties.”
Sudhinaraset, Ling, To, Melo, & Quach., 2017	“Assess the psychosocial needs and health status of Asian and Pacific Islander undocumented young adults, [and] guided by social capital theory, this qualitative study describes the social context of API undocumented young adults, including community and government perceptions, and how social relationships influence health.”
Sudhinaraset, To, Ling, Melo, & Chavarin., 2017	“Examines the influence of DACA on the health [mental health services/access] of API undocumented young adults.”
Ro & Van Hook., 2021	“Compare health patterns between Asians and Latinos by immigration status, [and examine] self-rated health, disability, and current health insurance.”
Ruhnke et al., 2022	“Investigates whether the commonly observed immigrant health advantage [self-rated health, chronic health condition, and obesity] persists among undocumented immigrants in the U.S. and provides nationally representative evidence on the health of this vulnerable population.”
Ling & Zhou, 2016	“Explore the extent to which legal status affects immigrants’ labor market performance and health status [self-rated health and health services/access]”
Woofter et al., 2022	“Examine differences in barriers to and discrimination in healthcare by DACA status [of young undocumented Latinx and Asian immigrants].”
Gao et al., 2016	“Explore Chinese immigrant restaurant workers’ perception of the U.S. health care system and their interactions with the health care system in interpreting meanings of health.”
Sudhinaraset, Choi, Nwankwo, & Young., 2022	“Quantify undocumented immigrants’ lifetime exposure to various immigration enforcement tactics and their association with delays in COVID-19 testing and healthcare behaviors.”
Sudhinaraset, Nwankwo, & Choi., 2022	“Examine the associations between immigration enforcement exposure and [COVID-19] vaccine intentions among undocumented immigrants in California.”
Sudhinaraset, Choi, Nakphone, Woofter, & Brindis., 2022	“Examine the association between DACA status and contraceptive use among undocumented young adults.”
Huang et al., 2021	“Explore [the links between migrant smuggling and its impact on the risk of infectious diseases, including HIV, for [Asian Pacific Americans who were smuggled into the U.S.]”

^a^Direct quotes are presented with quotation marks and with some verb tense changes

*API* Asian and Pacific Islander, *DACA* Deferred Action for Childhood Arrivals

### Research Aims

We identified five main themes across studies: DACA (n = 4), comparisons based only on status or race/ethnicity (n = 3), immigration enforcement (n = 2), social ties and social capital (n = 2), and studies on specific subpopulations (n = 2).

DACA was a recurring theme across four studies on different health outcomes. These four studies come from BRAVE datasets. The first quantitative study (n = 174) investigated whether DACA was protective against depression in an undocumented Asian and Pacific Islander (API) sample, and whether immigration enforcement experiences mediated this relationship [58]. A qualitative study (n = 32) examined how DACA influenced the social determinants of undocumented API in order to improve their mental health and well-being [29]. Another quantitative study (n = 203) of undocumented Asian and Latinx young adults investigated how DACA status was associated with barriers to health services and discrimination [63]. The final quantitative study (n = 204) compared the association between DACA and contraceptive use (defined as unprotected sex) in a sample of undocumented Asian and Latinx young adults.

A second theme was comparing UAIs to those with different immigration statuses or to other undocumented immigrants. A secondary data analysis using the Survey of Income and Program Participation (SIPP) (n = 31,300) examined the self-rated health, disability, and health insurance coverage of UAIs and undocumented Latinx participants [61]. In this study, researchers compared UAIs to their U.S-born Asian counterparts. A quantitative study of low-skilled Chinese immigrants in New York City (n = 372) investigated the impact of immigration status on self-rated health, likelihood of health care utilization, and employment outcomes [59]. A secondary data analysis (n = 435,528) using the 2000–2018 National Health Interview Survey (NHIS) and 2004, 2008, and 2014 cohorts of the SIPP  examined differences in self-rated health, chronic health conditions, and obesity between different U.S.-born and foreign-born groups [60]. With respect to UAIs, this analysis compared UAIs to undocumented immigrants from Mexico/Central America.

Understanding how immigration enforcement impacts UAIs was a third theme. There were two quantitative BRAVE studies which compared how levels of immigration enforcement interactions were associated with different COVID-related outcomes in samples of undocumented Latinx and Asian participants. The first study (n = 366) explored COVID-19 vaccine intentions as the main outcomes [64]; the second study (n = 326) explored COVID-19 positive tests and delayed testing and treatment as the main outcomes [65].

A fourth theme was studies on the role of social ties and social capital. A quantitative study (n = 143) examined (1) correlates of two types of social ties, bonding and bridging, and (2) associations between these ties and depression in a sample of API undocumented young adults [30]. A second qualitative study (n = 32) used social capital theory to assess the psychosocial needs and mental health of API undocumented young adults [28]. Both of these studies were from BRAVE datasets.

The remaining two qualitative studies were on specific subgroups of UAIs. One study conducted interviews (n = 11) with Asian and Pacific Americans who were living with HIV in New York City and Los Angeles [67]. All participants in this sample were smuggled into the U.S. and were undocumented at some point. Some participants were still UAIs, whereas some had gained legal status. The study aimed to understand these experiences of smuggling and the risk of infectious diseases. The last study was with Chinese immigrant restaurant workers, some of whom were UAIs, in an unspecific Midwestern state [62]. Using in-depth interviews (n = 18), researchers investigated how workers perceived the U.S. healthcare system and their interaction with health services and access.

### Study Characteristics

Table 2 presents detailed information on participant and study characteristics. The sample included nine quantitative studies [30, 58–61, 63–66] and four qualitative studies [28, 29, 62, 67]. All studies were cross-sectional or used pooled data. There were eleven studies which collected their own primary data and two studies which were secondary analyses of national datasets. Studies were geographically clustered. There were eight studies conducted in California, one conducted in an unspecified Midwestern state, one conducted in New York, and one conducted in both New York and California. BRAVE datasets were used by eight studies [57]. After reviewing the BRAVE publications, we concluded that at least three separate datasets were used in these papers. Qualitative studies had sample sizes ranging from 11 to 32. The sample size in quantitative studies where primary data were collected ranged from 143 to 372. Studies that collected their own data mainly recruited participants through non-probability sampling of in-person or web-based surveys, interviews, and focus group discussions. The two data analyses of secondary sources had large sample sizes of 31,300 and 435,528, respectively. Studies were conducted in different languages as follows: English only (n = 5), English and Spanish (n = 2), English, Mandarin, Cantonese, Japanese (n = 1), and Mandarin only (n = 1). There were four studies which did not specify the languages used. Although we limited the search to articles published in or after 2010, the time frames for the data collected were older. The oldest data were from Survey of Income and Program Participation (SIPP) and the National Health Interview Survey (NHIS) data from 2000. The most recent studies used data from 2021.

**Table 2:** Study Characteristics of the Articles in the Scoping Review

Study	Type	Time Frame	Source	Location	Sample	Total N	Ethnicities	Language	Health Issue
Manalo-Pedro et al., 2022	Quantitative	2019	Primary: BRAVE Studies Survey	California	Undocumented Asian and Pacific Islanders, ages 18–31, enrolled in college after June 15, 2012	174	Not specified	English	Depression
Ro, Nakphong, Choi, Nguyen, & Sudhinaraset., 2021	Quantitative	2019	Primary: BRAVE Studies Survey	California	Undocumented Asian and Pacific Islander, ages 18–31, enrolled in college after June 15, 2012	143	Chinese (n = 39), Korean (n = 21), Filipino (n = 12), Vietnamese (n = 9), Other (n = 52)	English	Depression
Sudhinaraset, Ling, To, Melo, & Quach., 2017	Qualitative	2015–2016	Primary: BRAVE Studies Focus Groups and In-depth Interviews	California	Undocumented Asian and Pacific Islanders, ages 18–31	32	South Korean (n = 11), Filipino (n = 7), Chinese (n = 2), Indonesian (n = 2), Other Asian/Pacific Islander (n = 6), Other Non-Asian (n = 4)	English	Psychosocial needs and health services/access
Sudhinaraset, To, Ling, Melo, & Chavarin., 2017	Qualitative	2015–2016	Primary: BRAVE Studies Focus Groups and In-depth Interviews	California	Undocumented Asian and Pacific Islanders, ages 18–31	32	South Korean (n = 11), Filipino (n = 7), Chinese (n = 2), Indonesian (n = 2), Other Asian/Pacific Islander (n = 6), Other Non-Asian (n = 4)	English	Mental health and health services/access
Ro & Van Hook., 2021	Quantitative	2001, 2004, 2008	Secondary: Restricted SIPP	Nationwide	Nationally representative Native and Foreign-born Latinx and Asian populations	31,300	Not specified	Not specified	Self-rated health, disability, and current health insurance
Ruhnke et al., 2022	Quantitative	2000–2018	Secondary: NHIS and SIPP	Nationwide	Nationally representative IPUMS and NHIS respondents	435,528	Not specified	Not specified	Self-rated health, chronic health condition [diabetes, hypertension, any heart conditions, asthma, chronic bronchitis, any form of cancer] and obesity
Liang and Zhou, 2016	Quantitative	2004	Primary: Survey	New York	Chinese immigrants in New York or with migratory experience to New York	372	Chinese only	Not specified	Self-rated health and health services/access
Woofter et al., 2022	Quantitative	2017	Primary: BRAVE Studies Internet-based Survey	California	Latinx and Asian undocumented immigrant young adults	203	Chinese (n = 18), Japanese (n = 12), and Taiwanese (n = 12) and Others not specified (n = 63)	Not specified	Health services/access
Gao et al., 2016	Qualitative	2011–2012	Primary: Interviews	Midwest, unspecified	Chinese immigrant restaurant workers	18	Chinese only	Mandarin	Health services/access
Sudhinaraset, Choi, Nwankwo, & Young., 2022	Quantitative	2020–2021	Primary: BRAVE Studies Internet-based Survey	California	Undocumented Asian and Latinx, ages 18–39	326	Not specified	English and Spanish	COVID-19
Sudhinaraset, Nwankwo, & Choi., 2022	Quantitative	2020–2021	Primary: BRAVE Studies Internet-based Survey	California	Undocumented Asian and Latinx, ages 18–39	326	Not specified	English and Spanish	COVID-19
Sudhinaraset, Choi, Nakphone, Woofter, & Brindis., 2022	Quantitative	2017	Primary: BRAVE Studies Internet-based Survey	California	Undocumented Asian and Latinx, ages 18–31	204	Chinese (n = 21), Japanese (n = 12), Korean (n = 11), Taiwanese (n = 9), and Others not specified (n = 52)	English	Contraceptive use
Huang et al., 2021	Qualitative	2017–2020	Primary: Interviews	New York and California	Asian or Pacific American immigrant adults, who entered the U.S. via smuggling and living with HIV	Not specified	Chinese (n = 7), Malaysian (n = 2), Vietnamese (n = 2)	English, Mandarin, Cantonese, Japanese	HIV

*NHIS* National Health Interview Survey, *SIPP* Survey of Income and Program Participation, *IPUMS* Integrated Public Use Microdata Series, *BRAVE* Building community Raising API Voices for health Equity

Based on the information provided in the articles, none had a fully UAI sample. We identified four studies with undocumented Asian participants and undocumented Pacific Islanders, collectively referred to as undocumented APIs.  [28–30, 58]. An additional four studies included UAIs or undocumented APIs and undocumented Latinx participants [63–66]. Likewise, three studies had UAIs and Asian immigrants with other types of immigration status [59, 62, 67]. The two studies with national samples included both non-Asian participants and documented participants [60, 61]. Studies with a large DACA sample included younger participants, aged 18–39 years old. All 13 studies focused on adults; however, none focused specifically on older adults or an aging population. Among all studies, eight studies explicitly included the ethnicity of participants, and five studies did not. Among those studies that included the ethnicities of participants, Chinese appeared to be the most common ethnic group. Table 2 has the sample size breakdown by ethnic subgroup for studies which included this information. Other ethnicities represented were Korean/South Korean, Japanese, Taiwanese, Filipino, Vietnamese, Malaysian, and Indonesian. There were two studies focused only on Chinese immigrants. Given the small sample size of studies, variability in topics studied and heterogeneity of comparison groups, drawing conclusions about the health status of UAIs was beyond the scope of this paper.

## Discussion

In this scoping review of the existing health literature of undocumented Asian immigrants in the U.S., we identified 13 peer-reviewed articles. Findings highlight several knowledge gaps in our understanding of the health of UAIs and delineate avenues for future research. The overall literature on the health and health experiences of UAIs lacks both topical depth and methodologic diversity. Studies were mixed in their focus, and provided evidence on mental health outcomes, self-rated health, health services and access, COVID-19, contraceptive use, HIV, and the effects of DACA. The study designs skewed slightly more quantitative, and many were concentrated geographically in California, where there are larger populations of UAIs. Undocumented immigrants from China were the most commonly studied Asian ethnic subgroup.

We identified eight articles from the same data source (BRAVE studies) and from California. This indicates that a small number of key research teams and datasets are driving research on the health of UAIs, which limits the generalizability of the results. However, ongoing projects such as Research on Immigrant Health and State Policy (RIGHTS) Study and the California Health Interview Survey (CHIS) are part of efforts to expand the study of UAI health. A review of the National Institutes of Health database identified at least two ongoing federally funded research projects that may include UAIs: one on the impacts of COVID-19 on Southeast Asian Americans and one on reproductive care among Asian immigrant women. This evidence suggests that research interest in UAI is burgeoning, and there will likely be more scholarship in the future.

Existing research was inconsistent in identifying the appropriate comparison group for research on UAIs. The differences in comparison groups made it difficult to equate studies. Moving forward, researchers are urged to carefully consider which comparison group may be most appropriate for their research question. For example, researchers interested in examining legal status stratification within Asian communities should aim to compare UAIs with other Asians immigrants (e.g., those who are documented) and U.S.-born Asians. On the other hand, researchers interested in understanding how racism and racialization impact UAIs could consider studying UAI experiences relative to undocumented White immigrants. Additionally, descriptive studies are also needed. Such descriptive epidemiologic studies [68] can provide valuable insights on UAI health and experiences.

Researchers must also diversify the breadth and depth of health outcomes studied. The small number of studies precluded us from making inferences about the health status of UAIs. Although we identified two studies on depression, more research on common mental health conditions such as depression and anxiety is warranted. These conditions might be highly prevalent among UAIs due to the stress associated with being undocumented [69]. Similarly, only one study examined chronic health conditions such as diabetes, hypertension, heart conditions, asthma, bronchitis, and cancer. It would be beneficial for more studies to explore these types of health conditions to understand the distribution among UAIs and highlight risk and protective factors. Researchers should continue to explore these questions with different UAI samples to ensure the replicability of findings. It is worth exploring how patterns of morbidity might intersect with UAI’s racialization and lack of legal status to produce disparities.

Our review only identified one study concerning the reproductive health of UAIs. Specifically, this study was on unprotected sex. Future research should explore how UAIs’ legal vulnerability might impact other facets of reproductive care, such as access to routine gynecologic care, contraceptives, and prenatal care. Access to safe abortion care for UAIs might be another area of research that is particularly salient since the U.S. Supreme Court’s decision on *Dobbs v. Jackson Women’s Health Organization* (2022).

Although eight studies identified the ethnicities of UAIs in their samples, they were most often unable to disaggregate results at this level due to small sample sizes. A majority of studies included participants who were younger, English-speaking, and more educated. We did not find studies that specifically focused on older undocumented adults, UAI women, or UAIs from minoritized religions. Asian immigrants are linguistically, educationally, culturally, and socioeconomically diverse. It stands to reason that research is unable to identify important concerns for specific subgroups of UAIs because of the lack of available data with robust sample sizes. This is especially important because while Asian immigrants, including UAIs, might look healthier as a whole, data aggregation might be masking more marginalized Asian subgroups who are at greater risk for poor health outcomes. This would also be salient when undocumented Pacific Islanders are included in the samples, such as in the BRAVE studies. Data disaggregation could help capture vulnerabilities that may be unique to undocumented Pacific Islanders. Although collecting more granular data can be challenging, several promising strategies have been proposed. These include oversampling Asian populations, increasing funding for research with Asian samples, developing community-based health registries, providing bilingual interpreters and translators, and pooling and linking existing datasets [70]. This supports movements to disaggregate research with Asian immigrant populations [41, 70]. Potential existing datasets to leverage could be Survey of Income and Program Participation (SIPP) and California Health Interview Survey (CHIS). In addition, researchers are encouraged to employ community-based and community-centered research methods. In the current political climate, with increasing xenophobic rhetoric, it can be challenging to engage undocumented participants. These methods can build on the relationships with trusted community-based organizations and young adults, who could serve as critical social brokers. These can help with the recruitment of this hard-to-reach population and allow researchers to conduct research with more diverse samples, to be able to generalize findings more broadly.

Notably, we did not find studies focusing on the health consequences of losing documentation status. A majority of recent UAIs become undocumented by losing their legal status. Future research must better understand the health consequences of losing status and access to resources– this might be particularly salient for UAIs who come to the U.S. as adults and who may not have a strong network of support to rely on. This review identified two studies on social capital and social ties. The protective effects of social capital have been documented with other samples of Asian immigrants [71, 72]. More research on the types and strengths of social ties within UAIs is an important area of study worth exploring. Regarding study design, we did not identify any longitudinal studies that captured the dynamic nature of immigration status. Immigration status, including undocumented status, can change over time. Future research should attend to how undocumented status might shift over time along the documentation continuum. Existing work has demonstrated that the length of time spent waiting for a more secure status, such as lawful permanent residency, can have health implications [73, 74]. It would be important to study how this differs in diverse local, regional, and state-based policy and political environments across the country.

Alongside this, this review did not identify research specific to mixed-status families. Mixed-status families include those where at least one family member is undocumented. Studies with other undocumented groups have shown that mixed-status families face unique challenges and stressors as a consequence of the precarious legal status of undocumented family members [75, 76]. Given that many UAIs initially migrate to the U.S. seeking employment or educational opportunities, they may have U.S. citizen spouses and children. Researchers should investigate how UAIs in mixed-status families navigate health and healthcare access, and in what ways the negative health impacts of insecure legal status might spill over to other family members, such as U.S. citizen children.

Lastly, there is a need for more research on how structural determinants, including structural racism and xenophobia, might impact the health and well-being of UAIs. We did not identify any such studies. Structural racism can impact the health of immigrants through racialized immigration policy, disproportionate immigration enforcement and criminalization, and economic exploitation and disinvestment [39]. More research is needed to strengthen our understanding of the pathways through which structural racism impacts the health of UAIs. These must consider how simultaneous experiences of racism, xenophobia, and other forms of discrimination intersect with UAIs vulnerable legal status to affect health outcomes, healthcare access, and health behaviors. Research in these domains would benefit from being firmly grounded in theoretical frameworks such as Intersectionality Theory that explicitly attend to these issues [39, 40, 77].

## Limitations

Although *PRISMA-ScR* guidelines [54] were followed and reference lists were hand-searched, it is still possible that we did not find all eligible studies published. Research not published in English was also excluded; hence eligible studies in other languages may have been missed. It is important to note that our review was limited to studies that explicitly focused on health. Therefore, studies with UAIs that provided perspectives on other outcomes, such as identity formation or educational outcomes, were not included. Although UAIs might have been present in other studies with undocumented populations or with Asian immigrants, we could not include such studies if they did not explicitly specify their presence in the sample population. Despite these limitations, we believe this is one of the first papers to systematically summarize the literature on the health of UAIs and findings underscore areas for further research.

## Conclusions

In this scoping review, we describe the nature and extent of existing peer-reviewed literature on the health of UAIs. Our findings suggest that this is a small but promising research domain. Studies with robust methodologies, larger and more diverse sample sizes, and more topical breadth are warranted. Results from this review can serve as a foundation for scholars interested in expanding research on the health of UAIs to develop strategies to increase positive health outcomes and reduce healthcare barriers for this population.
